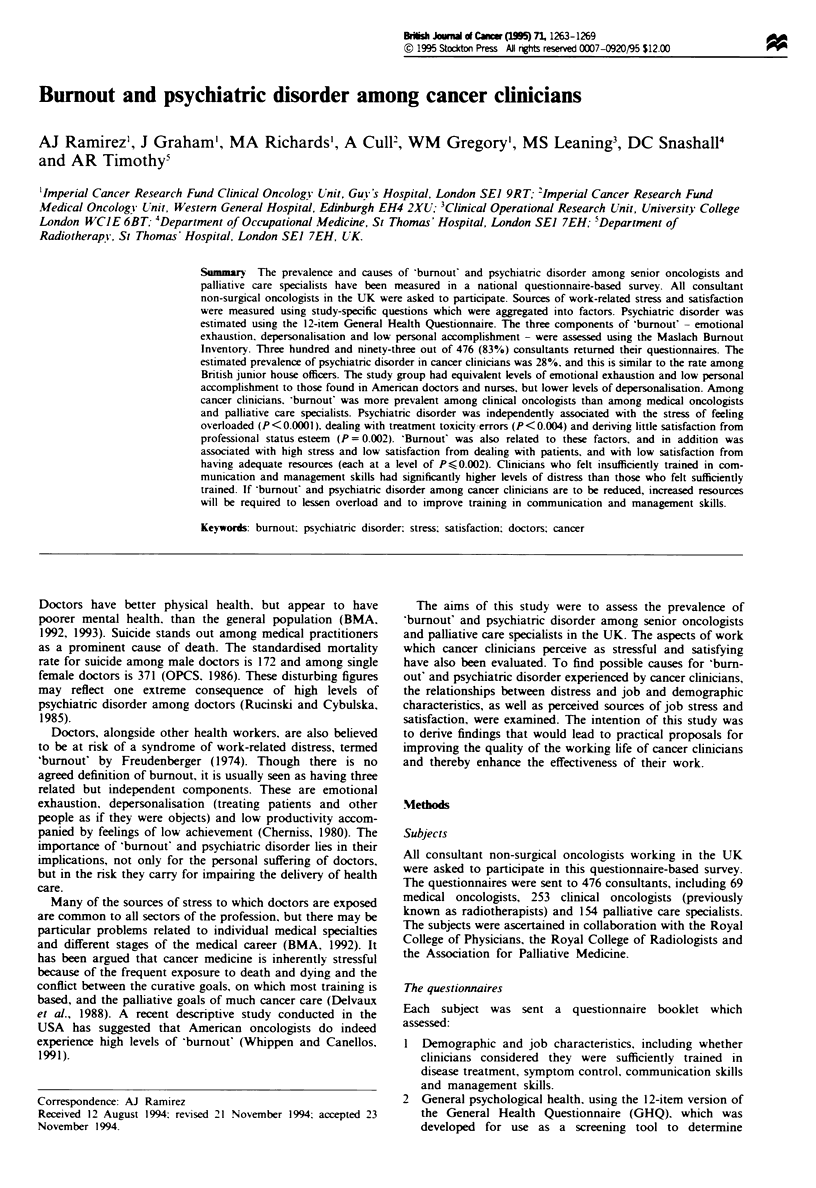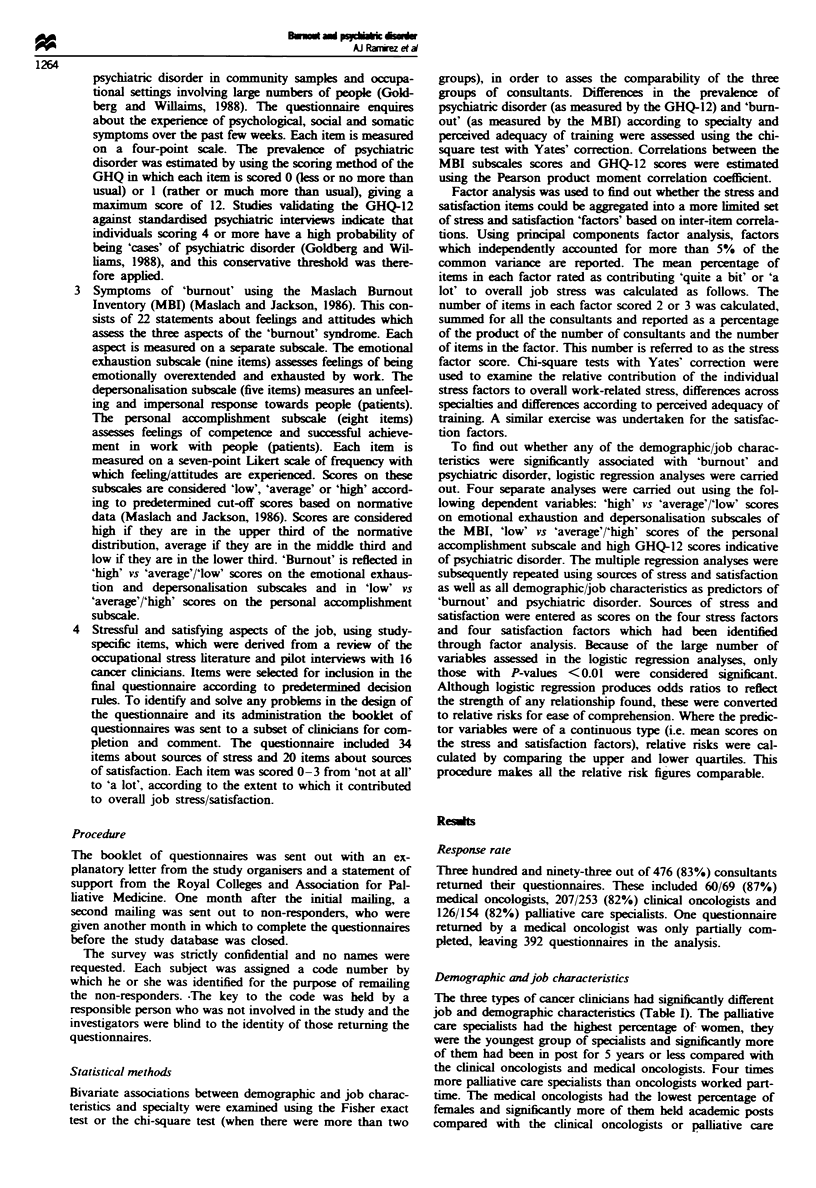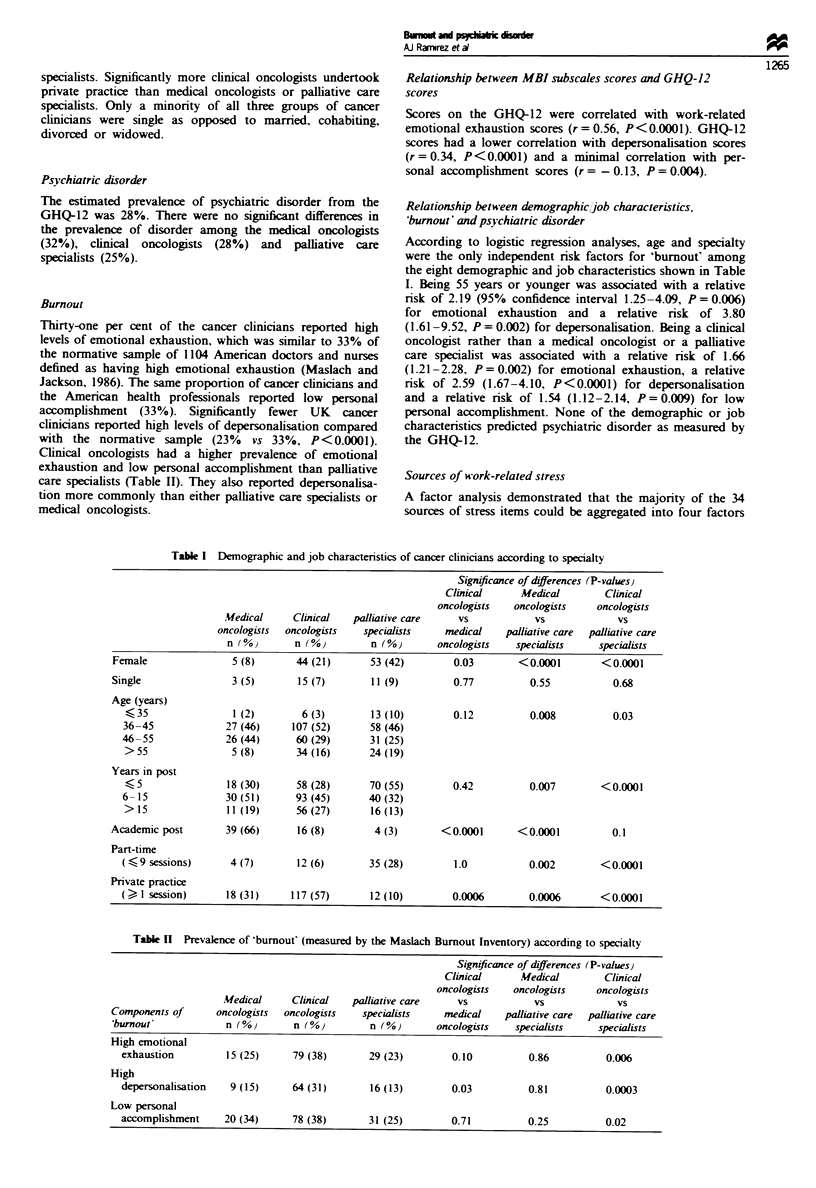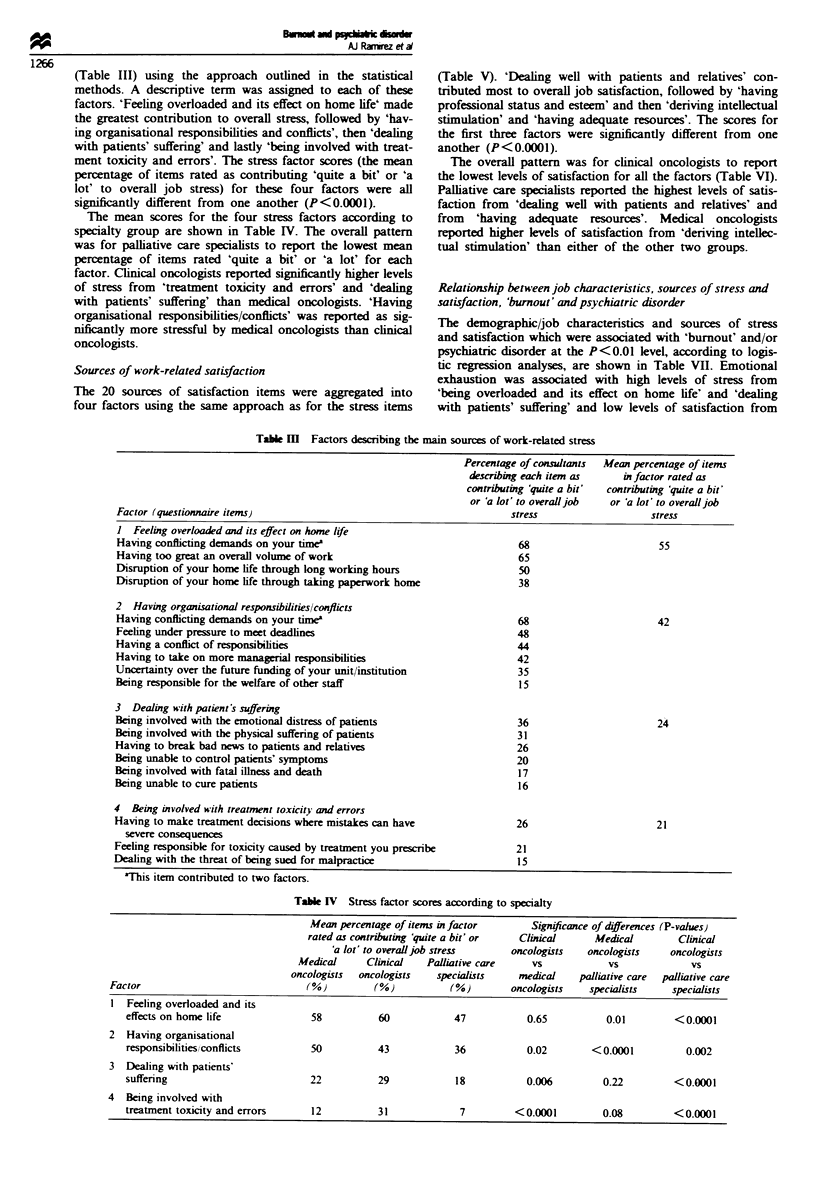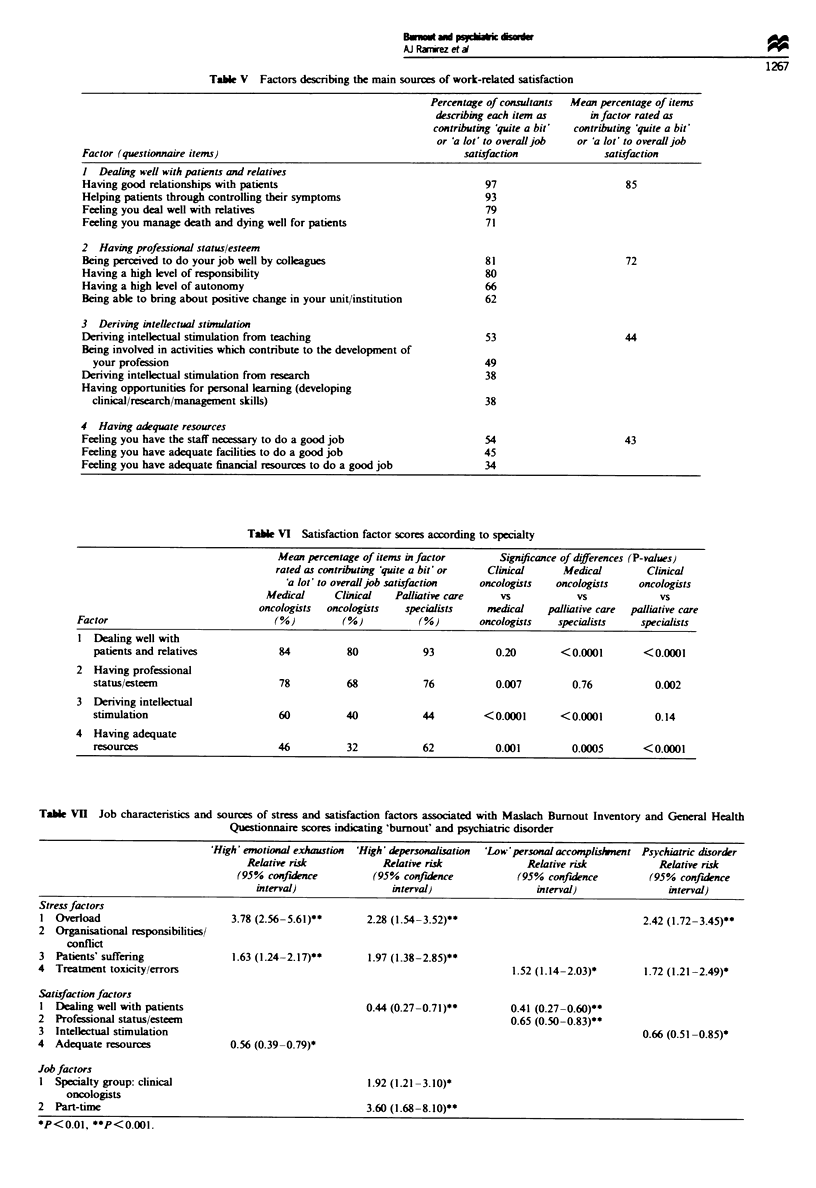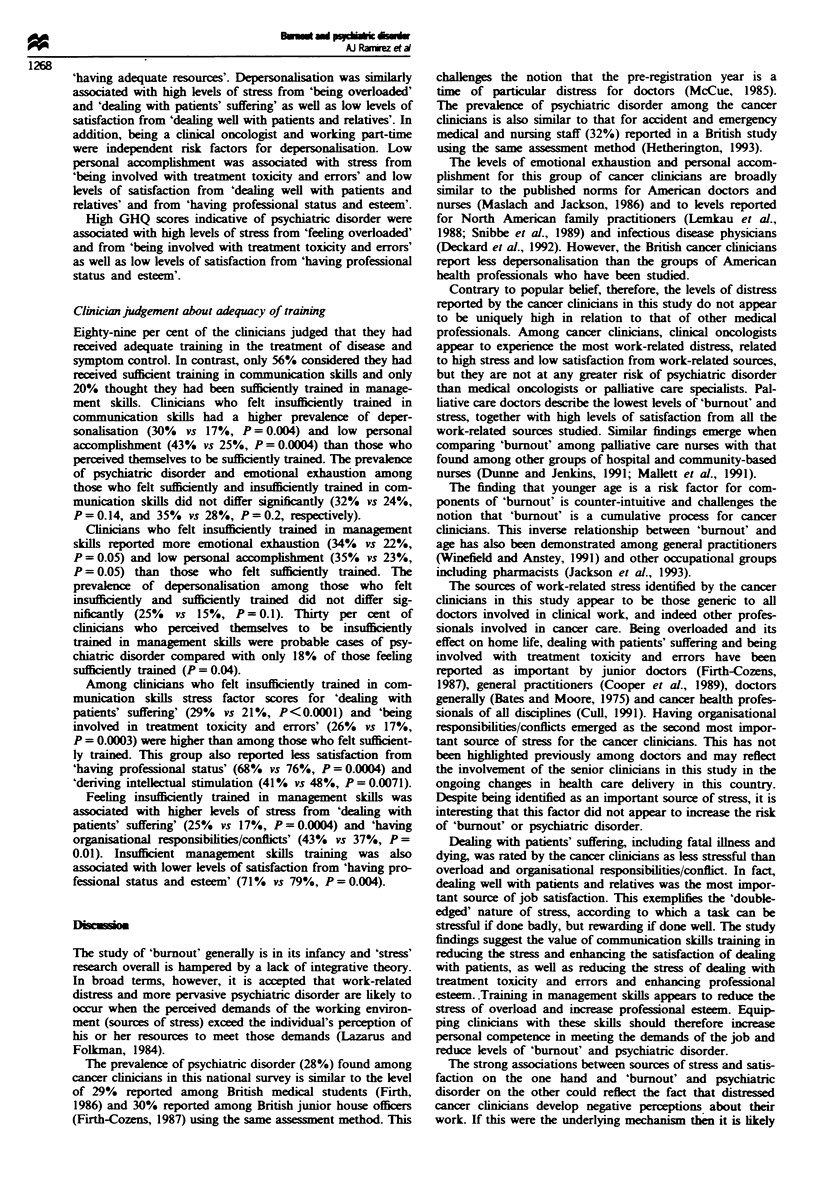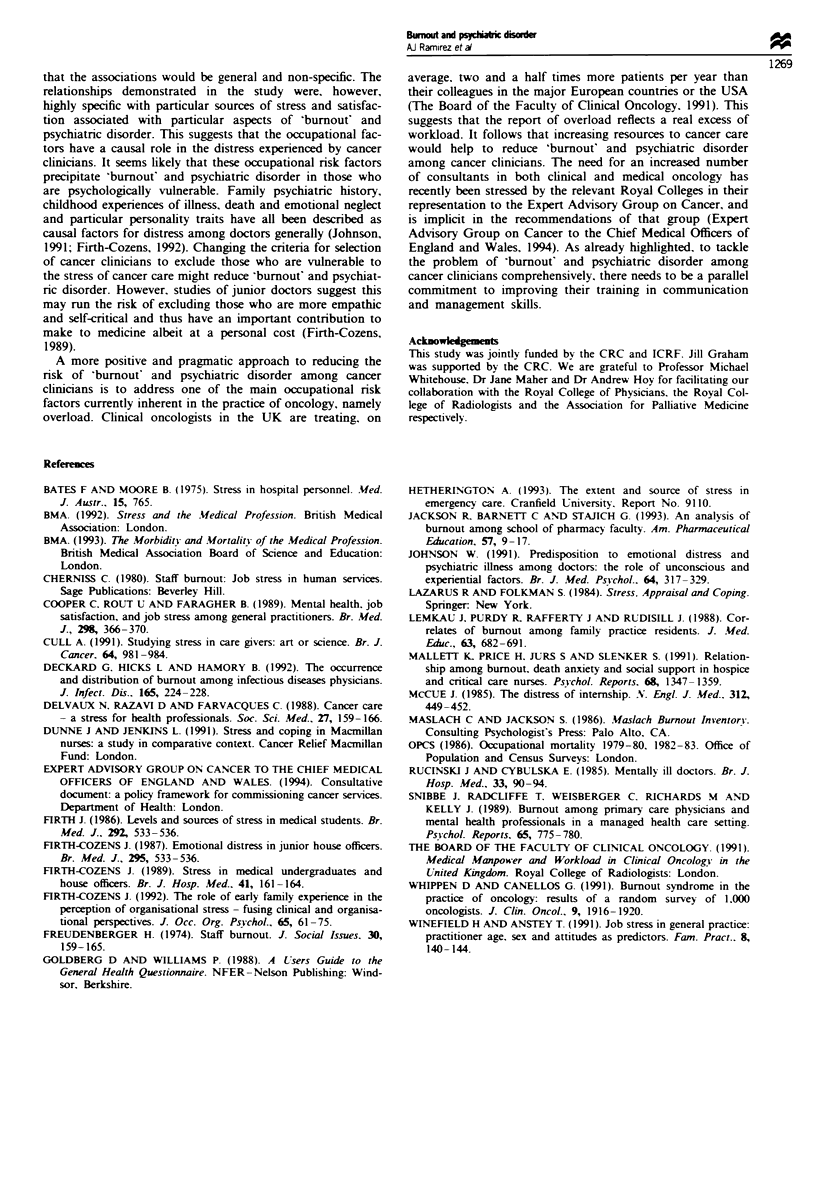# Burnout and psychiatric disorder among cancer clinicians.

**DOI:** 10.1038/bjc.1995.244

**Published:** 1995-06

**Authors:** A. J. Ramirez, J. Graham, M. A. Richards, A. Cull, W. M. Gregory, M. S. Leaning, D. C. Snashall, A. R. Timothy

**Affiliations:** Imperial Cancer Research Fund Clinical Oncology Unit, Guy's Hospital, London, UK.

## Abstract

The prevalence and causes of 'burnout' and psychiatric disorder among senior oncologists and palliative care specialists have been measured in a national questionnaire-based survey. All consultant non-surgical oncologists in the UK were asked to participate. Sources of work-related stress and satisfaction were measured using study-specific questions which were aggregated into factors. Psychiatric disorder was estimated using the 12-item General Health Questionnaire. The three components of 'burnout'--emotional exhaustion, depersonalisation and low personal accomplishment--were assessed using the Maslach Burnout Inventory. Three hundred and ninety-three out of 476 (83%) consultants returned their questionnaires. The estimated prevalence of psychiatric disorder in cancer clinicians was 28%, and this is similar to the rate among British junior house officers. The study group had equivalent levels of emotional exhaustion and low personal accomplishment to those found in American doctors and nurses, but lower levels of depersonalisation. Among cancer clinicians, 'burnout' was more prevalent among clinical oncologists than among medical oncologists and palliative care specialists. Psychiatric disorder was independently associated with the stress of feeling overloaded (P < 0.0001), dealing with treatment toxicity/errors (P < 0.004) and deriving little satisfaction from professional status/esteem (P = 0.002). 'Burnout' was also related to these factors, and in addition was associated with high stress and low satisfaction from dealing with patients, and with low satisfaction from having adequate resources (each at a level of P < or = 0.002). Clinicians who felt insufficiently trained in communication and management skills had significantly higher levels of distress than those who felt sufficiently trained. If 'burnout' and psychiatric disorder among cancer clinicians are to be reduced, increased resources will be required to lessen overload and to improve training in communication and management skills.


© 1995 Stockton Press All rights reserved 0007-0920/95 $12.00

### Burnout and psychiatric disorder among cancer clinicians

AJ Ramirez[1], J Graham[1], MA Richards[1], A Cull[2], WM Gregory[1], MS Leaning[3], DC Snashall[4] and AR Timothy[5]

[1]Imperial Cancer Research Fund Clinical Oncology Unit, Guy's Hospital, London SE1 9RT; [2]Imperial Cancer Research Fund Medical Oncology Unit, Western General Hospital, Edinburgh EH4 2XU; [3]Clinical Operational Research Unit, University College London WC1E 6BT; [4]Department of Occupational Medicine, St Thomas' Hospital, London SE1 7EH; [5]Department of Radiotherapy, St Thomas' Hospital, London SE1 7EH, UK.

**Summary** The prevalence and causes of 'burnout' and psychiatric disorder among senior oncologists and palliative care specialists have been measured in a national questionnaire-based survey. All consultant non-surgical oncologists in the UK were asked to participate. Sources of work-related stress and satisfaction were measured using study-specific questions which were aggregated into factors. Psychiatric disorder was estimated using the 12-item General Health Questionnaire. The three components of 'burnout' – emotional exhaustion, depersonalisation and low personal accomplishment – were assessed using the Maslach Burnout Inventory. Three hundred and ninety-three out of 476 (83%) consultants returned their questionnaires. The estimated prevalence of psychiatric disorder in cancer clinicians was 28%, and this is similar to the rate among British junior house officers. The study group had equivalent levels of emotional exhaustion and low personal accomplishment to those found in American doctors and nurses, but lower levels of depersonalisation. Among cancer clinicians, 'burnout' was more prevalent among clinical oncologists than among medical oncologists and palliative care specialists. Psychiatric disorder was independently associated with the stress of feeling overloaded ($P<0.0001$), dealing with treatment toxicity/errors ($P<0.004$) and deriving little satisfaction from professional status esteem ($P=0.002$). 'Burnout' was also related to these factors, and in addition was associated with high stress and low satisfaction from dealing with patients, and with low satisfaction from having adequate resources (each at a level of $P \leqslant 0.002$). Clinicians who felt insufficiently trained in communication and management skills had significantly higher levels of distress than those who felt sufficiently trained. If 'burnout' and psychiatric disorder among cancer clinicians are to be reduced, increased resources will be required to lessen overload and to improve training in communication and management skills.

**Keywords**: burnout; psychiatric disorder; stress; satisfaction; doctors; cancer

Doctors have better physical health, but appear to have poorer mental health, than the general population (BMA, 1992, 1993). Suicide stands out among medical practitioners as a prominent cause of death. The standardised mortality rate for suicide among male doctors is 172 and among single female doctors is 371 (OPCS, 1986). These disturbing figures may reflect one extreme consequence of high levels of psychiatric disorder among doctors (Rucinski and Cybulska, 1985).

Doctors, alongside other health workers, are also believed to be at risk of a syndrome of work-related distress, termed 'burnout' by Freudenberger (1974). Though there is no agreed definition of burnout, it is usually seen as having three related but independent components. These are emotional exhaustion, depersonalisation (treating patients and other people as if they were objects) and low productivity accompanied by feelings of low achievement (Cherniss, 1980). The importance of 'burnout' and psychiatric disorder lies in their implications, not only for the personal suffering of doctors, but in the risk they carry for impairing the delivery of health care.

Many of the sources of stress to which doctors are exposed are common to all sectors of the profession, but there may be particular problems related to individual medical specialties and different stages of the medical career (BMA, 1992). It has been argued that cancer medicine is inherently stressful because of the frequent exposure to death and dying and the conflict between the curative goals, on which most training is based, and the palliative goals of much cancer care (Delvaux *et al.*, 1988). A recent descriptive study conducted in the USA has suggested that American oncologists do indeed experience high levels of 'burnout' (Whippen and Canellos, 1991).

Correspondence: AJ Ramirez
Received 12 August 1994; revised 21 November 1994; accepted 23 November 1994.

The aims of this study were to assess the prevalence of 'burnout' and psychiatric disorder among senior oncologists and palliative care specialists in the UK. The aspects of work which cancer clinicians perceive as stressful and satisfying have also been evaluated. To find possible causes for 'burnout' and psychiatric disorder experienced by cancer clinicians, the relationships between distress and job and demographic characteristics, as well as perceived sources of job stress and satisfaction, were examined. The intention of this study was to derive findings that would lead to practical proposals for improving the quality of the working life of cancer clinicians and thereby enhance the effectiveness of their work.

### Methods

### *Subjects*

All consultant non-surgical oncologists working in the UK were asked to participate in this questionnaire-based survey. The questionnaires were sent to 476 consultants, including 69 medical oncologists, 253 clinical oncologists (previously known as radiotherapists) and 154 palliative care specialists. The subjects were ascertained in collaboration with the Royal College of Physicians, the Royal College of Radiologists and the Association for Palliative Medicine.

### *The questionnaires*

Each subject was sent a questionnaire booklet which assessed:

1 Demographic and job characteristics, including whether clinicians considered they were sufficiently trained in disease treatment, symptom control, communication skills and management skills.
2 General psychological health, using the 12-item version of the General Health Questionnaire (GHQ), which was developed for use as a screening tool to determine psychiatric disorder in community samples and occupational settings involving large numbers of people (Goldberg and Willaims, 1988). The questionnaire enquires about the experience of psychological, social and somatic symptoms over the past few weeks. Each item is measured on a four-point scale. The prevalence of psychiatric disorder was estimated by using the scoring method of the GHQ in which each item is scored 0 (less or no more than usual) or 1 (rather or much more than usual), giving a maximum score of 12. Studies validating the GHQ-12 against standardised psychiatric interviews indicate that individuals scoring 4 or more have a high probability of being 'cases' of psychiatric disorder (Goldberg and Williams, 1988), and this conservative threshold was therefore applied.

3 Symptoms of 'burnout' using the Maslach Burnout Inventory (MBI) (Maslach and Jackson, 1986). This consists of 22 statements about feelings and attitudes which assess the three aspects of the 'burnout' syndrome. Each aspect is measured on a separate subscale. The emotional exhaustion subscale (nine items) assesses feelings of being emotionally overextended and exhausted by work. The depersonalisation subscale (five items) measures an unfeeling and impersonal response towards people (patients). The personal accomplishment subscale (eight items) assesses feelings of competence and successful achievement in work with people (patients). Each item is measured on a seven-point Likert scale of frequency with which feeling/attitudes are experienced. Scores on these subscales are considered 'low', 'average' or 'high' according to predetermined cut-off scores based on normative data (Maslach and Jackson, 1986). Scores are considered high if they are in the upper third of the normative distribution, average if they are in the middle third and low if they are in the lower third. 'Burnout' is reflected in 'high' *vs* 'average'/'low' scores on the emotional exhaustion and depersonalisation subscales and in 'low' *vs* 'average'/'high' scores on the personal accomplishment subscale.

4 Stressful and satisfying aspects of the job, using study-specific items, which were derived from a review of the occupational stress literature and pilot interviews with 16 cancer clinicians. Items were selected for inclusion in the final questionnaire according to predetermined decision rules. To identify and solve any problems in the design of the questionnaire and its administration the booklet of questionnaires was sent to a subset of clinicians for completion and comment. The questionnaire included 34 items about sources of stress and 20 items about sources of satisfaction. Each item was scored 0–3 from 'not at all' to 'a lot', according to the extent to which it contributed to overall job stress/satisfaction.

### *Procedure*

The booklet of questionnaires was sent out with an explanatory letter from the study organisers and a statement of support from the Royal Colleges and Association for Palliative Medicine. One month after the initial mailing, a second mailing was sent out to non-responders, who were given another month in which to complete the questionnaires before the study database was closed.

The survey was strictly confidential and no names were requested. Each subject was assigned a code number by which he or she was identified for the purpose of remailing the non-responders. The key to the code was held by a responsible person who was not involved in the study and the investigators were blind to the identity of those returning the questionnaires.

### *Statistical methods*

Bivariate associations between demographic and job characteristics and specialty were examined using the Fisher exact test or the chi-square test (when there were more than two groups), in order to asses the comparability of the three groups of consultants. Differences in the prevalence of psychiatric disorder (as measured by the GHQ-12) and 'burnout' (as measured by the MBI) according to specialty and perceived adequacy of training were assessed using the chi-square test with Yates' correction. Correlations between the MBI subscales scores and GHQ-12 scores were estimated using the Pearson product moment correlation coefficient.

Factor analysis was used to find out whether the stress and satisfaction items could be aggregated into a more limited set of stress and satisfaction 'factors' based on inter-item correlations. Using principal components factor analysis, factors which independently accounted for more than 5% of the common variance are reported. The mean percentage of items in each factor rated as contributing 'quite a bit' or 'a lot' to overall job stress was calculated as follows. The number of items in each factor scored 2 or 3 was calculated, summed for all the consultants and reported as a percentage of the product of the number of consultants and the number of items in the factor. This number is referred to as the stress factor score. Chi-square tests with Yates' correction were used to examine the relative contribution of the individual stress factors to overall work-related stress, differences across specialties and differences according to perceived adequacy of training. A similar exercise was undertaken for the satisfaction factors.

To find out whether any of the demographic/job characteristics were significantly associated with 'burnout' and psychiatric disorder, logistic regression analyses were carried out. Four separate analyses were carried out using the following dependent variables: 'high' *vs* 'average'/'low' scores on emotional exhaustion and depersonalisation subscales of the MBI, 'low' *vs* 'average'/'high' scores of the personal accomplishment subscale and high GHQ-12 scores indicative of psychiatric disorder. The multiple regression analyses were subsequently repeated using sources of stress and satisfaction as well as all demographic/job characteristics as predictors of 'burnout' and psychiatric disorder. Sources of stress and satisfaction were entered as scores on the four stress factors and four satisfaction factors which had been identified through factor analysis. Because of the large number of variables assessed in the logistic regression analyses, only those with $P$-values $<0.01$ were considered significant. Although logistic regression produces odds ratios to reflect the strength of any relationship found, these were converted to relative risks for ease of comprehension. Where the predictor variables were of a continuous type (i.e. mean scores on the stress and satisfaction factors), relative risks were calculated by comparing the upper and lower quartiles. This procedure makes all the relative risk figures comparable.

### Results

### *Response rate*

Three hundred and ninety-three out of 476 (83%) consultants returned their questionnaires. These included 60/69 (87%) medical oncologists, 207/253 (82%) clinical oncologists and 126/154 (82%) palliative care specialists. One questionnaire returned by a medical oncologist was only partially completed, leaving 392 questionnaires in the analysis.

### *Demographic and job characteristics*

The three types of cancer clinicians had significantly different job and demographic characteristics (Table I). The palliative care specialists had the highest percentage of women, they were the youngest group of specialists and significantly more of them had been in post for 5 years or less compared with the clinical oncologists and medical oncologists. Four times more palliative care specialists than oncologists worked part-time. The medical oncologists had the lowest percentage of females and significantly more of them held academic posts compared with the clinical oncologists or palliative care specialists. Significantly more clinical oncologists undertook private practice than medical oncologists or palliative care specialists. Only a minority of all three groups of cancer clinicians were single as opposed to married, cohabiting, divorced or widowed.

*Psychiatric disorder*

The estimated prevalence of psychiatric disorder from the GHQ-12 was 28%. There were no significant differences in the prevalence of disorder among the medical oncologists (32%), clinical oncologists (28%) and palliative care specialists (25%).

*Burnout*

Thirty-one per cent of the cancer clinicians reported high levels of emotional exhaustion, which was similar to 33% of the normative sample of 1104 American doctors and nurses defined as having high emotional exhaustion (Maslach and Jackson, 1986). The same proportion of cancer clinicians and the American health professionals reported low personal accomplishment (33%). Significantly fewer UK cancer clinicians reported high levels of depersonalisation compared with the normative sample (23% *vs* 33%, $P<0.0001$). Clinical oncologists had a higher prevalence of emotional exhaustion and low personal accomplishment than palliative care specialists (Table II). They also reported depersonalisation more commonly than either palliative care specialists or medical oncologists.

*Relationship between MBI subscales scores and GHQ-12 scores*

Scores on the GHQ-12 were correlated with work-related emotional exhaustion scores ($r=0.56$, $P<0.0001$). GHQ-12 scores had a lower correlation with depersonalisation scores ($r=0.34$, $P<0.0001$) and a minimal correlation with personal accomplishment scores ($r=-0.13$, $P=0.004$).

*Relationship between demographic/job characteristics, 'burnout' and psychiatric disorder*

According to logistic regression analyses, age and specialty were the only independent risk factors for 'burnout' among the eight demographic and job characteristics shown in Table I. Being 55 years or younger was associated with a relative risk of 2.19 (95% confidence interval 1.25–4.09, $P=0.006$) for emotional exhaustion and a relative risk of 3.80 (1.61–9.52, $P=0.002$) for depersonalisation. Being a clinical oncologist rather than a medical oncologist or a palliative care specialist was associated with a relative risk of 1.66 (1.21–2.28, $P=0.002$) for emotional exhaustion, a relative risk of 2.59 (1.67–4.10, $P<0.0001$) for depersonalisation and a relative risk of 1.54 (1.12–2.14, $P=0.009$) for low personal accomplishment. None of the demographic or job characteristics predicted psychiatric disorder as measured by the GHQ-12.

*Sources of work-related stress*

A factor analysis demonstrated that the majority of the 34 sources of stress items could be aggregated into four factors

**Table I** Demographic and job characteristics of cancer clinicians according to specialty

| | | | | Significance of differences (P-values) | | |
|---|---|---|---|---|---|---|
| | *Medical oncologists* n (%) | *Clinical oncologists* n (%) | *palliative care specialists* n (%) | *Clinical oncologists vs medical oncologists* | *Medical oncologists vs palliative care specialists* | *Clinical oncologists vs palliative care specialists* |
| Female | 5 (8) | 44 (21) | 53 (42) | 0.03 | <0.0001 | <0.0001 |
| Single | 3 (5) | 15 (7) | 11 (9) | 0.77 | 0.55 | 0.68 |
| Age (years) | | | | | | |
| ≤35 | 1 (2) | 6 (3) | 13 (10) | 0.12 | 0.008 | 0.03 |
| 36–45 | 27 (46) | 107 (52) | 58 (46) | | | |
| 46–55 | 26 (44) | 60 (29) | 31 (25) | | | |
| >55 | 5 (8) | 34 (16) | 24 (19) | | | |
| Years in post | | | | | | |
| ≤5 | 18 (30) | 58 (28) | 70 (55) | 0.42 | 0.007 | <0.0001 |
| 6–15 | 30 (51) | 93 (45) | 40 (32) | | | |
| >15 | 11 (19) | 56 (27) | 16 (13) | | | |
| Academic post | 39 (66) | 16 (8) | 4 (3) | <0.0001 | <0.0001 | 0.1 |
| Part-time (≤9 sessions) | 4 (7) | 12 (6) | 35 (28) | 1.0 | 0.002 | <0.0001 |
| Private practice (≥1 session) | 18 (31) | 117 (57) | 12 (10) | 0.0006 | 0.0006 | <0.0001 |

**Table II** Prevalence of 'burnout' (measured by the Maslach Burnout Inventory) according to specialty

| | | | | Significance of differences (P-values) | | |
|---|---|---|---|---|---|---|
| *Components of 'burnout'* | *Medical oncologists* n (%) | *Clinical oncologists* n (%) | *palliative care specialists* n (%) | *Clinical oncologists vs medical oncologists* | *Medical oncologists vs palliative care specialists* | *Clinical oncologists vs palliative care specialists* |
| High emotional exhaustion | 15 (25) | 79 (38) | 29 (23) | 0.10 | 0.86 | 0.006 |
| High depersonalisation | 9 (15) | 64 (31) | 16 (13) | 0.03 | 0.81 | 0.0003 |
| Low personal accomplishment | 20 (34) | 78 (38) | 31 (25) | 0.71 | 0.25 | 0.02 |

(Table III) using the approach outlined in the statistical methods. A descriptive term was assigned to each of these factors. 'Feeling overloaded and its effect on home life' made the greatest contribution to overall stress, followed by 'having organisational responsibilities and conflicts', then 'dealing with patients' suffering' and lastly 'being involved with treatment toxicity and errors'. The stress factor scores (the mean percentage of items rated as contributing 'quite a bit' or 'a lot' to overall job stress) for these four factors were all significantly different from one another ($P<0.0001$).

The mean scores for the four stress factors according to specialty group are shown in Table IV. The overall pattern was for palliative care specialists to report the lowest mean percentage of items rated 'quite a bit' or 'a lot' for each factor. Clinical oncologists reported significantly higher levels of stress from 'treatment toxicity and errors' and 'dealing with patients' suffering' than medical oncologists. 'Having organisational responsibilities/conflicts' was reported as significantly more stressful by medical oncologists than clinical oncologists.

*Sources of work-related satisfaction*

The 20 sources of satisfaction items were aggregated into four factors using the same approach as for the stress items (Table V). 'Dealing well with patients and relatives' contributed most to overall job satisfaction, followed by 'having professional status and esteem' and then 'deriving intellectual stimulation' and 'having adequate resources'. The scores for the first three factors were significantly different from one another ($P<0.0001$).

The overall pattern was for clinical oncologists to report the lowest levels of satisfaction for all the factors (Table VI). Palliative care specialists reported the highest levels of satisfaction from 'dealing well with patients and relatives' and from 'having adequate resources'. Medical oncologists reported higher levels of satisfaction from 'deriving intellectual stimulation' than either of the other two groups.

*Relationship between job characteristics, sources of stress and satisfaction, 'burnout' and psychiatric disorder*

The demographic/job characteristics and sources of stress and satisfaction which were associated with 'burnout' and/or psychiatric disorder at the $P<0.01$ level, according to logistic regression analyses, are shown in Table VII. Emotional exhaustion was associated with high levels of stress from 'being overloaded and its effect on home life' and 'dealing with patients' suffering' and low levels of satisfaction from

**Table III** Factors describing the main sources of work-related stress

| *Factor (questionnaire items)* | *Percentage of consultants describing each item as contributing 'quite a bit' or 'a lot' to overall job stress* | *Mean percentage of items in factor rated as contributing 'quite a bit' or 'a lot' to overall job stress* |
|---|---|---|
| *1 Feeling overloaded and its effect on home life* | | |
| Having conflicting demands on your time[a] | 68 | 55 |
| Having too great an overall volume of work | 65 | |
| Disruption of your home life through long working hours | 50 | |
| Disruption of your home life through taking paperwork home | 38 | |
| *2 Having organisational responsibilities/conflicts* | | |
| Having conflicting demands on your time[a] | 68 | 42 |
| Feeling under pressure to meet deadlines | 48 | |
| Having a conflict of responsibilities | 44 | |
| Having to take on more managerial responsibilities | 42 | |
| Uncertainty over the future funding of your unit/institution | 35 | |
| Being responsible for the welfare of other staff | 15 | |
| *3 Dealing with patient's suffering* | | |
| Being involved with the emotional distress of patients | 36 | 24 |
| Being involved with the physical suffering of patients | 31 | |
| Having to break bad news to patients and relatives | 26 | |
| Being unable to control patients' symptoms | 20 | |
| Being involved with fatal illness and death | 17 | |
| Being unable to cure patients | 16 | |
| *4 Being involved with treatment toxicity and errors* | | |
| Having to make treatment decisions where mistakes can have severe consequences | 26 | 21 |
| Feeling responsible for toxicity caused by treatment you prescribe | 21 | |
| Dealing with the threat of being sued for malpractice | 15 | |

[a]This item contributed to two factors.

**Table IV** Stress factor scores according to specialty

| Factor | *Mean percentage of items in factor rated as contributing 'quite a bit' or 'a lot' to overall job stress* | | | *Significance of differences (P-values)* | | |
|---|---|---|---|---|---|---|
| | *Medical oncologists (%)* | *Clinical oncologists (%)* | *Palliative care specialists (%)* | *Clinical oncologists vs medical oncologists* | *Medical oncologists vs palliative care specialists* | *Clinical oncologists vs palliative care specialists* |
| 1 Feeling overloaded and its effects on home life | 58 | 60 | 47 | 0.65 | 0.01 | <0.0001 |
| 2 Having organisational responsibilities/conflicts | 50 | 43 | 36 | 0.02 | <0.0001 | 0.002 |
| 3 Dealing with patients' suffering | 22 | 29 | 18 | 0.006 | 0.22 | <0.0001 |
| 4 Being involved with treatment toxicity and errors | 12 | 31 | 7 | <0.0001 | 0.08 | <0.0001 |

Table V Factors describing the main sources of work-related satisfaction

| Factor (questionnaire items) | Percentage of consultants describing each item as contributing 'quite a bit' or 'a lot' to overall job satisfaction | Mean percentage of items in factor rated as contributing 'quite a bit' or 'a lot' to overall job satisfaction |
|---|---|---|
| *1 Dealing well with patients and relatives* | | |
| Having good relationships with patients | 97 | 85 |
| Helping patients through controlling their symptoms | 93 | |
| Feeling you deal well with relatives | 79 | |
| Feeling you manage death and dying well for patients | 71 | |
| *2 Having professional status/esteem* | | |
| Being perceived to do your job well by colleagues | 81 | 72 |
| Having a high level of responsibility | 80 | |
| Having a high level of autonomy | 66 | |
| Being able to bring about positive change in your unit/institution | 62 | |
| *3 Deriving intellectual stimulation* | | |
| Deriving intellectual stimulation from teaching | 53 | 44 |
| Being involved in activities which contribute to the development of your profession | 49 | |
| Deriving intellectual stimulation from research | 38 | |
| Having opportunities for personal learning (developing clinical/research/management skills) | 38 | |
| *4 Having adequate resources* | | |
| Feeling you have the staff necessary to do a good job | 54 | 43 |
| Feeling you have adequate facilities to do a good job | 45 | |
| Feeling you have adequate financial resources to do a good job | 34 | |

Table VI Satisfaction factor scores according to specialty

| Factor | Mean percentage of items in factor rated as contributing 'quite a bit' or 'a lot' to overall job satisfaction | | | Significance of differences (P-values) | | |
|---|---|---|---|---|---|---|
| | Medical oncologists (%) | Clinical oncologists (%) | Palliative care specialists (%) | Clinical oncologists vs medical oncologists | Medical oncologists vs palliative care specialists | Clinical oncologists vs palliative care specialists |
| 1 Dealing well with patients and relatives | 84 | 80 | 93 | 0.20 | <0.0001 | <0.0001 |
| 2 Having professional status/esteem | 78 | 68 | 76 | 0.007 | 0.76 | 0.002 |
| 3 Deriving intellectual stimulation | 60 | 40 | 44 | <0.0001 | <0.0001 | 0.14 |
| 4 Having adequate resources | 46 | 32 | 62 | 0.001 | 0.0005 | <0.0001 |

Table VII Job characteristics and sources of stress and satisfaction factors associated with Maslach Burnout Inventory and General Health Questionnaire scores indicating 'burnout' and psychiatric disorder

| | 'High' emotional exhaustion Relative risk (95% confidence interval) | 'High' depersonalisation Relative risk (95% confidence interval) | 'Low' personal accomplishment Relative risk (95% confidence interval) | Psychiatric disorder Relative risk (95% confidence interval) |
|---|---|---|---|---|
| *Stress factors* | | | | |
| 1 Overload | 3.78 (2.56–5.61)** | 2.28 (1.54–3.52)** | | 2.42 (1.72–3.45)** |
| 2 Organisational responsibilities/ conflict | | | | |
| 3 Patients' suffering | 1.63 (1.24–2.17)** | 1.97 (1.38–2.85)** | | |
| 4 Treatment toxicity/errors | | | 1.52 (1.14–2.03)* | 1.72 (1.21–2.49)* |
| *Satisfaction factors* | | | | |
| 1 Dealing well with patients | | 0.44 (0.27–0.71)** | 0.41 (0.27–0.60)** | |
| 2 Professional status/esteem | | | 0.65 (0.50–0.83)** | |
| 3 Intellectual stimulation | | | | 0.66 (0.51–0.85)* |
| 4 Adequate resources | 0.56 (0.39–0.79)* | | | |
| *Job factors* | | | | |
| 1 Specialty group: clinical oncologists | | 1.92 (1.21–3.10)* | | |
| 2 Part-time | | 3.60 (1.68–8.10)** | | |

$*P<0.01$, $**P<0.001$.

'having adequate resources'. Depersonalisation was similarly associated with high levels of stress from 'being overloaded' and 'dealing with patients' suffering' as well as low levels of satisfaction from 'dealing well with patients and relatives'. In addition, being a clinical oncologist and working part-time were independent risk factors for depersonalisation. Low personal accomplishment was associated with stress from 'being involved with treatment toxicity and errors' and low levels of satisfaction from 'dealing well with patients and relatives' and from 'having professional status and esteem'.

High GHQ scores indicative of psychiatric disorder were associated with high levels of stress from 'feeling overloaded' and from 'being involved with treatment toxicity and errors' as well as low levels of satisfaction from 'having professional status and esteem'.

*Clinician judgement about adequacy of training*

Eighty-nine per cent of the clinicians judged that they had received adequate training in the treatment of disease and symptom control. In contrast, only 56% considered they had received sufficient training in communication skills and only 20% thought they had been sufficiently trained in management skills. Clinicians who felt insufficiently trained in communication skills had a higher prevalence of depersonalisation (30% *vs* 17%, $P = 0.004$) and low personal accomplishment (43% *vs* 25%, $P = 0.0004$) than those who perceived themselves to be sufficiently trained. The prevalence of psychiatric disorder and emotional exhaustion among those who felt sufficiently and insufficiently trained in communication skills did not differ significantly (32% *vs* 24%, $P = 0.14$, and 35% *vs* 28%, $P = 0.2$, respectively).

Clinicians who felt insufficiently trained in management skills reported more emotional exhaustion (34% *vs* 22%, $P = 0.05$) and low personal accomplishment (35% *vs* 23%, $P = 0.05$) than those who felt sufficiently trained. The prevalence of depersonalisation among those who felt insufficiently and sufficiently trained did not differ significantly (25% *vs* 15%, $P = 0.1$). Thirty per cent of clinicians who perceived themselves to be insufficiently trained in management skills were probable cases of psychiatric disorder compared with only 18% of those feeling sufficiently trained ($P = 0.04$).

Among clinicians who felt insufficiently trained in communication skills stress factor scores for 'dealing with patients' suffering' (29% *vs* 21%, $P < 0.0001$) and 'being involved in treatment toxicity and errors' (26% *vs* 17%, $P = 0.0003$) were higher than among those who felt sufficiently trained. This group also reported less satisfaction from 'having professional status' (68% *vs* 76%, $P = 0.0004$) and 'deriving intellectual stimulation (41% *vs* 48%, $P = 0.0071$).

Feeling insufficiently trained in management skills was associated with higher levels of stress from 'dealing with patients' suffering' (25% *vs* 17%, $P = 0.0004$) and 'having organisational responsibilities/conflicts' (43% *vs* 37%, $P = 0.01$). Insufficient management skills training was also associated with lower levels of satisfaction from 'having professional status and esteem' (71% *vs* 79%, $P = 0.004$).

### Discussion

The study of 'burnout' generally is in its infancy and 'stress' research overall is hampered by a lack of integrative theory. In broad terms, however, it is accepted that work-related distress and more pervasive psychiatric disorder are likely to occur when the perceived demands of the working environment (sources of stress) exceed the individual's perception of his or her resources to meet those demands (Lazarus and Folkman, 1984).

The prevalence of psychiatric disorder (28%) found among cancer clinicians in this national survey is similar to the level of 29% reported among British medical students (Firth, 1986) and 30% reported among British junior house officers (Firth-Cozens, 1987) using the same assessment method. This challenges the notion that the pre-registration year is a time of particular distress for doctors (McCue, 1985). The prevalence of psychiatric disorder among the cancer clinicians is also similar to that for accident and emergency medical and nursing staff (32%) reported in a British study using the same assessment method (Hetherington, 1993).

The levels of emotional exhaustion and personal accomplishment for this group of cancer clinicians are broadly similar to the published norms for American doctors and nurses (Maslach and Jackson, 1986) and to levels reported for North American family practitioners (Lemkau *et al.*, 1988; Snibbe *et al.*, 1989) and infectious disease physicians (Deckard *et al.*, 1992). However, the British cancer clinicians report less depersonalisation than the groups of American health professionals who have been studied.

Contrary to popular belief, therefore, the levels of distress reported by the cancer clinicians in this study do not appear to be uniquely high in relation to that of other medical professionals. Among cancer clinicians, clinical oncologists appear to experience the most work-related distress, related to high stress and low satisfaction from work-related sources, but they are not at any greater risk of psychiatric disorder than medical oncologists or palliative care specialists. Palliative care doctors describe the lowest levels of 'burnout' and stress, together with high levels of satisfaction from all the work-related sources studied. Similar findings emerge when comparing 'burnout' among palliative care nurses with that found among other groups of hospital and community-based nurses (Dunne and Jenkins, 1991; Mallett *et al.*, 1991).

The finding that younger age is a risk factor for components of 'burnout' is counter-intuitive and challenges the notion that 'burnout' is a cumulative process for cancer clinicians. This inverse relationship between 'burnout' and age has also been demonstrated among general practitioners (Winefield and Anstey, 1991) and other occupational groups including pharmacists (Jackson *et al.*, 1993).

The sources of work-related stress identified by the cancer clinicians in this study appear to be those generic to all doctors involved in clinical work, and indeed other professionals involved in cancer care. Being overloaded and its effect on home life, dealing with patients' suffering and being involved with treatment toxicity and errors have been reported as important by junior doctors (Firth-Cozens, 1987), general practitioners (Cooper *et al.*, 1989), doctors generally (Bates and Moore, 1975) and cancer health professionals of all disciplines (Cull, 1991). Having organisational responsibilities/conflicts emerged as the second most important source of stress for the cancer clinicians. This has not been highlighted previously among doctors and may reflect the involvement of the senior clinicians in this study in the ongoing changes in health care delivery in this country. Despite being identified as an important source of stress, it is interesting that this factor did not appear to increase the risk of 'burnout' or psychiatric disorder.

Dealing with patients' suffering, including fatal illness and dying, was rated by the cancer clinicians as less stressful than overload and organisational responsibilities/conflict. In fact, dealing well with patients and relatives was the most important source of job satisfaction. This exemplifies the 'double-edged' nature of stress, according to which a task can be stressful if done badly, but rewarding if done well. The study findings suggest the value of communication skills training in reducing the stress and enhancing the satisfaction of dealing with patients, as well as reducing the stress of dealing with treatment toxicity and errors and enhancing professional esteem. Training in management skills appears to reduce the stress of overload and increase professional esteem. Equipping clinicians with these skills should therefore increase personal competence in meeting the demands of the job and reduce levels of 'burnout' and psychiatric disorder.

The strong associations between sources of stress and satisfaction on the one hand and 'burnout' and psychiatric disorder on the other could reflect the fact that distressed cancer clinicians develop negative perceptions about their work. If this were the underlying mechanism then it is likely that the associations would be general and non-specific. The relationships demonstrated in the study were, however, highly specific with particular sources of stress and satisfaction associated with particular aspects of 'burnout' and psychiatric disorder. This suggests that the occupational factors have a causal role in the distress experienced by cancer clinicians. It seems likely that these occupational risk factors precipitate 'burnout' and psychiatric disorder in those who are psychologically vulnerable. Family psychiatric history, childhood experiences of illness, death and emotional neglect and particular personality traits have all been described as causal factors for distress among doctors generally (Johnson, 1991; Firth-Cozens, 1992). Changing the criteria for selection of cancer clinicians to exclude those who are vulnerable to the stress of cancer care might reduce 'burnout' and psychiatric disorder. However, studies of junior doctors suggest this may run the risk of excluding those who are more empathic and self-critical and thus have an important contribution to make to medicine albeit at a personal cost (Firth-Cozens, 1989).

A more positive and pragmatic approach to reducing the risk of 'burnout' and psychiatric disorder among cancer clinicians is to address one of the main occupational risk factors currently inherent in the practice of oncology, namely overload. Clinical oncologists in the UK are treating, on average, two and a half times more patients per year than their colleagues in the major European countries or the USA (The Board of the Faculty of Clinical Oncology, 1991). This suggests that the report of overload reflects a real excess of workload. It follows that increasing resources to cancer care would help to reduce 'burnout' and psychiatric disorder among cancer clinicians. The need for an increased number of consultants in both clinical and medical oncology has recently been stressed by the relevant Royal Colleges in their representation to the Expert Advisory Group on Cancer, and is implicit in the recommendations of that group (Expert Advisory Group on Cancer to the Chief Medical Officers of England and Wales, 1994). As already highlighted, to tackle the problem of 'burnout' and psychiatric disorder among cancer clinicians comprehensively, there needs to be a parallel commitment to improving their training in communication and management skills.

**Acknowledgements**
This study was jointly funded by the CRC and ICRF. Jill Graham was supported by the CRC. We are grateful to Professor Michael Whitehouse, Dr Jane Maher and Dr Andrew Hoy for facilitating our collaboration with the Royal College of Physicians, the Royal College of Radiologists and the Association for Palliative Medicine respectively.

**References**

BATES F AND MOORE B. (1975). Stress in hospital personnel. *Med. J. Austr.*, **15**, 765.
BMA. (1992). *Stress and the Medical Profession*. British Medical Association: London.
BMA. (1993). *The Morbidity and Mortality of the Medical Profession*. British Medical Association Board of Science and Education: London.
CHERNISS C. (1980). Staff burnout: Job stress in human services. Sage Publications: Beverley Hill.
COOPER C, ROUT U AND FARAGHER B. (1989). Mental health, job satisfaction, and job stress among general practitioners. *Br. Med. J.*, **298**, 366-370.
CULL A. (1991). Studying stress in care givers: art or science. *Br. J. Cancer*, **64**, 981-984.
DECKARD G, HICKS L AND HAMORY B. (1992). The occurrence and distribution of burnout among infectious diseases physicians. *J. Infect. Dis.*, **165**, 224-228.
DELVAUX N, RAZAVI D AND FARVACQUES C. (1988). Cancer care - a stress for health professionals. *Soc. Sci. Med.*, **27**, 159-166.
DUNNE J AND JENKINS L. (1991). Stress and coping in Macmillan nurses: a study in comparative context. Cancer Relief Macmillan Fund: London.
EXPERT ADVISORY GROUP ON CANCER TO THE CHIEF MEDICAL OFFICERS OF ENGLAND AND WALES. (1994). Consultative document: a policy framework for commissioning cancer services. Department of Health: London.
FIRTH J. (1986). Levels and sources of stress in medical students. *Br. Med. J.*, **292**, 533-536.
FIRTH-COZENS J. (1987). Emotional distress in junior house officers. *Br. Med. J.*, **295**, 533-536.
FIRTH-COZENS J. (1989). Stress in medical undergraduates and house officers. *Br. J. Hosp. Med.*, **41**, 161-164.
FIRTH-COZENS J. (1992). The role of early family experience in the perception of organisational stress - fusing clinical and organisational perspectives. *J. Occ. Org. Psychol.*, **65**, 61-75.
FREUDENBERGER H. (1974). Staff burnout. *J. Social Issues*, **30**, 159-165.
GOLDBERG D AND WILLIAMS P. (1988). *A Users Guide to the General Health Questionnaire*. NFER-Nelson Publishing: Windsor, Berkshire.
HETHERINGTON A. (1993). The extent and source of stress in emergency care. Cranfield University, Report No. 9110.
JACKSON R, BARNETT C AND STAJICH G. (1993). An analysis of burnout among school of pharmacy faculty. *Am. Pharmaceutical Education*, **57**, 9-17.
JOHNSON W. (1991). Predisposition to emotional distress and psychiatric illness among doctors: the role of unconscious and experiential factors. *Br. J. Med. Psychol.*, **64**, 317-329.
LAZARUS R AND FOLKMAN S. (1984). *Stress, Appraisal and Coping*. Springer: New York.
LEMKAU J, PURDY R, RAFFERTY J AND RUDISILL J. (1988). Correlates of burnout among family practice residents. *J. Med. Educ.*, **63**, 682-691.
MALLETT K, PRICE H, JURS S AND SLENKER S. (1991). Relationship among burnout, death anxiety and social support in hospice and critical care nurses. *Psychol. Reports*, **68**, 1347-1359.
McCUE J. (1985). The distress of internship. *N. Engl. J. Med.*, **312**, 449-452.
MASLACH C AND JACKSON S. (1986). *Maslach Burnout Inventory*. Consulting Psychologist's Press: Palo Alto, CA.
OPCS (1986). Occupational mortality 1979-80, 1982-83. Office of Population and Census Surveys: London.
RUCINSKI J AND CYBULSKA E. (1985). Mentally ill doctors. *Br. J. Hosp. Med.*, **33**, 90-94.
SNIBBE J, RADCLIFFE T, WEISBERGER C, RICHARDS M AND KELLY J. (1989). Burnout among primary care physicians and mental health professionals in a managed health care setting. *Psychol. Reports*, **65**, 775-780.
THE BOARD OF THE FACULTY OF CLINICAL ONCOLOGY. (1991). *Medical Manpower and Workload in Clinical Oncology in the United Kingdom*. Royal College of Radiologists: London.
WHIPPEN D AND CANELLOS G. (1991). Burnout syndrome in the practice of oncology: results of a random survey of 1,000 oncologists. *J. Clin. Oncol.*, **9**, 1916-1920.
WINEFIELD H AND ANSTEY T. (1991). Job stress in general practice: practitioner age, sex and attitudes as predictors. *Fam. Pract.*, **8**, 140-144.